# The Interplay between Obstructive Sleep Apnea and Atrial Fibrillation

**DOI:** 10.3389/fneur.2017.00668

**Published:** 2017-12-11

**Authors:** Erika Marulanda-Londoño, Seemant Chaturvedi

**Affiliations:** ^1^Stroke Program, Department of Neurology, University of Miami Miller School of Medicine, Miami, FL, United States

**Keywords:** atrial fibrillation, arrhythmia, obstructive sleep apnea, cardiac monitoring, hypertension

## Abstract

Atrial fibrillation (AF) is the most common cardiac arrhythmia. Obstructive sleep apnea (OSA) is also an increasingly common condition. Both entities are risk factors for ischemic stroke and both conditions are linked with increased mortality. Mechanical effects of obesity and sleep apnea can lead to increased afterload, left ventricular hypertrophy, and left atrial fibrosis and remodeling. These changes can result in an increased risk of AF development. The current paper summarizes the evidence for the bidirectional relationship between AF and OSA. The merits of selective screening for these two conditions are also discussed.

## Introduction

Atrial fibrillation (AF) is the most prevalent cardiac arrhythmia and carries significant morbidity, mortality, and health care costs. Prevalence of AF increases with age and more than 1/3 of patients with AF are ≥80 years old. AF is associated with structural heart disease, extra-cardiac factors, including hypertension, diabetes, obesity, and sleep apnea among others (1). Obesity increases risk for AF, with a progressive increase according to body mass index (BMI) (2). AF can result in cerebral and systemic embolization and heart failure. Those with AF have a fivefold increase risk of stroke and twofold increase risk of cardiac-related death (1).

Obstructive sleep apnea (OSA) is the most common sleep-breathing disorder. It is characterized by collapse of the pharyngeal airway leading to repetitive interruption of ventilation during sleep. Diagnosis of OSA is usually made when a patient has an apnea–hypopnea index ≥5 and excessive daytime sleepiness. It is likely underdiagnosed and prevalence depends on stringency of definition used. Estimated prevalence is about 1 in 5 adults having at least mild OSA, and 1 in 15 having moderate to severe OSA (3). OSA has been associated with hypertension, heart failure, and AF. Other risk factors for OSA include older age and upper airway soft tissue abnormalities. The prevalence of OSA progressively increases as BMI increases (4, 5). Untreated severe OSA is associated with increased cardiovascular mortality of any cause (6, 7). OSA has been shown to increase the risk of stroke, independently of other traditional stroke risk factors, such as hypertension, diabetes, and AF (8).

Obstructive sleep apnea and AF share many common risk factors. The prevalence of both OSA and AF is rising likely due to increases in cardiovascular disease and obesity. The close association between cardiovascular disease and OSA, and cardiovascular disease and AF may obscure a directly causal relationship between OSA and AF. These chronic diseases are associated, and the interplay of their pathophysiology is complex and likely bidirectional. OSA may promote AF, and AF contributes to OSA development. Nevertheless, there is extensive evidence that these entities are linked, independently with other cardiovascular comorbidities. Importantly, both OSA and AF are risk factors for stroke and have known treatments.

Many trials show a significant absolute risk reduction of stroke secondary to AF with the use of anticoagulants. Anticoagulation is standard of care in patients with CHADS2 score ≥2 (9). Less is know about the degree of cardiovascular risk reduction with use of continuous positive airway pressure (CPAP). Studies focusing on CPAP treatment for primary and secondary prevention of cardiovascular disease in patients with OSA have used a wide range of inclusion criteria in terms of sleep apnea severity and symptomaticity, making it difficult to draw conclusions. While, some showed better cardiovascular outcomes among patients adherent to CPAP (≥4 h per night) vs. those not on CPAP or non-adherent, others have shown no effect (10–12). More recently, the Sleep Apnea cardioVascular Endpoints (SAVE) Trial focused on secondary prevention in adults with cardiovascular disease and OSA, and found that risk of serious cardiovascular events was not lower among those in the CPAP group compared to those who received usual care alone (13). Notably, patients in the CPAP arm of the trial were complaint on average for only 3.3 h, which many would consider noncompliance and does not meet standard of care. Thus, more studies are needed on the role of CPAP therapy in both primary and secondary prevention of stroke and cardiovascular disease.

## The Relationship Between OSA and AF

The pathophysiologic mechanisms of the association between AF and OSA are complex and likely multifactorial. Controversy over this association and its directionality exists, because of the high incidence of cardiovascular comorbidities in patients with OSA and AF. Patients with OSA have a higher incidence of AF than the general population.

An analysis from the Sleep Heart Study compared 228 with sleep-disordered breathing (SDB) and 338 without SDB to assess whether prevalence of AF and clinically significant ventricular arrhythmias would be increased in patients with SDB, even after adjusting for confounders (age, sex, BMI, coronary disease). Those with SDB had increased likelihood of AF (OR 4.01; 95% CI, 1.03–15.74), non-sustained ventricular tachycardia (OR 3.40; 95% CI, 1.02–11.20), and complex ventricular ectopy (OR 1.74; 95% CI, 1.11–2.74). Worsened severity of OSA increased risk for AF (14). A comparison of 151 patients with AF and 312 patients with no current or past AF showed that proportion of patients with OSA was significantly higher in the group with AF than in the non-AF group (49 vs. 32%, *p* = 0.0004). After adjustment for risk factors, including age, BMI, hypertension, and CHF, those with AF had doubled the odds of OSA (OR 2.19; 95% CI, 1.40–3.42) (15). Another analysis from the Sleep Heart Study looked at whether apneas and/or hypopneas were temporally associated with episodes of PAF or NSVT. They found that although rate of arrhythmia was low, there was a higher risk of arrhythmia (OR 17.5; 85% CI, 5.3–58.4) in the 90 s following a respiratory disturbance compared with following normal breathing. Median duration was 7 s for AF with the longest episode lasting 5 min (16). This supports a temporal link between SDB and arrhythmia, though it does not establish causality.

Most of the evidence suggests a strong association between OSA and AF. Animal studies have shown autonomic nervous system imbalances brought on by hypoxia as well as hypercapnia may precipitate electrical changes in atria that predispose to AF (17, 18). Studies in rats have shown that fluctuations in intrathoracic pressure resulted in cardiac structural remodeling with increased left atrial dilatation and increased fibrosis (19, 20).

Obstructive sleep apnea can cause changes in cardiac function and structure. Repetitive forced inspiration against a closed airway results in negative intrathoracic pressure that may result in increased cardiac afterload, increased atrial size and wall stress that can result in atrial remodeling that may predispose to arrhythmia. Severe intermittent hypoxemia, acidosis, and hypercapnia can result in autonomic dysfunction with sympathetic activation, heart rate elevation and depression, and blood pressure elevation. This can persist in daytime with normal oxygen levels, thus the effect may outlast the apneic event (3). Vasoactive substances such as endothelin, released in the setting of hypoxemia may lead to long-term damage of vessels and predisposition to hypertension (3). Hypoxemia may also trigger inflammation, as demonstrated by elevation of inflammatory markers such as C-reactive protein, in patients with OSA. Autonomic stimulation, inflammation and oxidative stress, and the renin–angiotensin–aldosterone system are also involved in increased arrhythmia susceptibility (1).

Obesity is likely one of the links between OSA and AF. Obesity is associated with risk of development of AF and OSA. A small study focused on patients evaluated for bariatric surgery (mean BMI of 47) found that 88% had obstructive sleep-related breathing disorders (upper airway resistance syndrome or OSA) and 71% had OSA (21). In terms of AF, a meta-analysis looked at five population cohort studies including 78,602 subjects and found that obese individuals had a 49% increased risk of developing AF compared to non-obese (RR 1.49; 95% CI, 1.36–1.64), and risk of AF increased with greater BMI (22). Obesity can lead to cardiac remodeling. Magnani et al. provide a potential mechanistic explanation for obesity as the mediator for all risk factors leading to AF. In their schematic, obesity, *via* metabolic correlates (hypertension, dyslipidemia, and insulin resistance), mechanical effects OSA, and raised intrathoracic pressure, coronary disease, ventricular adaptation (hypertrophy), and inflammation ultimately leads to atrial adaptation (raised atrial pressures and enlargement and altered electrical function) which results in AF (23).

## OSA, Stroke, and AF

Determining cause of stroke is crucial to reducing risk of a subsequent event. Once etiology is established, risk factors can be targeted and addressed accordingly. In cryptogenic stroke, in which an etiology has not been determined, it becomes difficult to know what to target. Assessing the relationship between OSA and stroke has been the focus of various studies. A retrospective case-control study involving 53 patients with and without OSA, and stroke within 1 year of polysomnography showed that those with OSA were more likely to have cardioembolic stroke (72 vs. 33%, *p* = 0.01). AF was more common in OSA patients (59 vs. 24%, *p* = 0.01) (24). A larger case control study examined the occurrence of AF in patients with OSA and stroke. It included 108 subjects, of which 34 had OSA based on polysomnography and index stroke in the 5-year study period. AF was more common in cases (50 vs. 11%, *p* < 0.01) and on adjusted analysis (controlling for age, BMI, coronary disease, hypertension, diabetes, hyperlipidemia, tobacco status) was associated with stroke (OR 5.34; 95% CI, 1.79–17.29) (25). These studies identified AF as a likely cause of stroke in OSA patients with stroke. The results emphasize that clinicians need to have a high level of suspicion for AF in patients with OSA and stroke.

## OSA, AF, and Risk of Stroke and Thromboembolism

For patients with AF, assessment of stroke and thromboembolism risk, and subsequent use of appropriate therapy is crucial in prevention of stroke. Risk stratification schemes for AF including CHADS_2_ and CHA_2_DS_2_-VASc use demographics and vascular risk factors to help inform risk of stroke as well as recommended treatment for stroke prevention. CHA_2_DS_2_-VASc is felt to better assess risk of stroke among patients with a CHADS_2_ of 0 or 1. These are the most widely used schemes and do not include OSA as a parameter. Given what is known about the interplay between OSA and AF, and OSA and stroke, some have considered using OSA as a variable to help inform risk of stroke and recommended treatment. One study examined hospitalized patients with primary diagnosis of AF to assess if those with coexisting OSA had a higher stroke risk based on CHADS_2_ and CHA_2_DS_2_-VASc scores. Forty-eight percent of patients (121/254) had OSA. They tended to be older, have a higher BMI, and were more likely to have history of hypertension, diabetes, and previous stroke. In the OSA group, median CHADS_2_ was 1.2 ± 0.9 (vs. 0.8 ± 0.6, *p* < 0.0001) and median CHA_2_DS_2_-VASc score was 2.2 ± 1.7 (vs. 1.5 ± 1.1, *p* = 0.001) (26). The results of this specific study do not prove that OSA is an independent risk factor for stroke in patients with AF, but can be a predictor of higher stroke risk overall. Further studies are needed to decide if OSA should be included in stroke risk predictive scales for AF.

## Impact of OSA Treatment on AF

Risk of recurrent AF after is diminished by OSA treatment with CPAP. The presence of untreated OSA in patients after cardioversion is associated with 82% risk of recurrence of AF within 1 year, which is double the rate seen in patients in whom OSA is treated effectively (27). Two studies focused on pulmonary vein isolation with radiofrequency ablation demonstrated that patients with untreated OSA had higher recurrence of AF after ablation (28, 29). In these studies, rate of recurrence among CPAP-treated individuals was similar to that of patients without OSA. This demonstrates that OSA plays some role in preservation of AF that can be mitigated by treatment. The Outcomes Registry for Better Informed Treatment of AF (ORBIT-AF) trial examined 10,132 patients and found that patients with OSA (18%) required more hospitalizations, but no increase in risk of overall or cardiovascular death when compared with patients without OSA. Patients with OSA on CPAP were less likely to progress to more permanent AF (HR 0.66; 95% CI, 0.46–0.94; *p* = 0.02). The mechanism for how CPAP therapy reduces risk for AF recurrence is not well established, but reversing cardiac structural changes caused by OSA may play a role. Additionally, CPAP may mitigate other AF risk factors such as hypertension and intrathoracic pressure changes.

## Is There a Benefit in Screening for AF in Patients with OSA?

The association between OSA and increased risk for AF has been established in previous studies. OSA is an independent risk factor for stroke, and AF is likely to play a role in the development of stroke in patients with OSA. AF is usually diagnosed after symptoms related to arrhythmia, such as palpitations, usually due to rapid ventricular response. About 18% of strokes caused by AF occur in patients in which the AF is a new diagnosis, discovered upon the evaluation for stroke etiology. Evidence for screening unselected asymptomatic patients for AF is weak. The known association between OSA and AF brings to mind the question of screening patients with high-risk conditions like OSA, for silent AF. The Screening Study for Undiagnosed AF (STUDY-AF) included 75 male subjects of age ≥55 and with ≥2 AF risk factors coronary disease, heart failure, hypertension, DM, and sleep apnea (central or obstructive) (30). Subjects were screened for arrhythmia in an outpatient setting for 2 weeks using a Zio wearable patch-based device. Thirty-three percent had sleep apnea (not specified if OSA or CSA). An AF episode was defined as the presence of ≥30 s of continuous AF during monitoring. AF was detected in four subjects (5.3%; all with CHADS_2_ ≥1 and CHA_2_DS_2_-VASc score ≥2).

The specific question of whether there is a benefit in screening patients with OSA for asymptomatic AF has not been systematically tackled. Early detection of AF in a patient population already at higher risk for stroke, such as in patients with OSA, could reduce burden of stroke as it could result in the initiation of stroke preventive therapies like anticoagulation. One small study by Chanda et al. looked at the feasibility and yield of screening for AF in patients with OSA (31). Twenty subjects with newly diagnosed severe OSA (AHI ≥ 30) and no previous history of AF were monitored for 7 days (prior to initiation of CPAP therapy). No clinically meaningful, silent AF (defined by authors as ≥5 min) was detected though one subject had AF lasting 7 s. Of note, the meaning of clinically significant duration is variable in different studies and thus, controversial.

Overall, studies focused on detection of silent AF in risk patients have been small, have had variable definitions of clinically meaningful AF duration, and had relatively short monitoring times. One consideration is that a longer monitoring period for AF would be useful. The Arrhythmia Detection in OSA (ADIOS) study is currently recruiting subjects with OSA (goal 200) to undergo 30-day cardiac monitoring using the Lifestar Act III device to monitor arrhythmia (ClinicalTrials.gov Identifier: NCT02743520). Primary stroke prevention for eligible patients with AF with anticoagulation is much less expensive than post stroke care. If this study shows that non-invasive screening for AF in OSA patients is feasible and effective, it could have a significant impact on public health.

## Is There a Benefit in Screening for OSA in Patients with AF?

Current recommendations for OSA screening are limited. Sensitivity and specificity of screening scales is not well established. The US Preventive Services Task Force (USPSTF) notes there is currently insufficient evidence to assess benefits or harm of use of screening tools or scales for OSA in asymptomatic individuals (32). The American Heart Association/American College of Cardiology/Heart Rhythm Society guidelines state that a sleep study may be useful if sleep apnea is suspected (1). Notably, the American Academy of Sleep Medicine considers AF patients (along with those with BMI >35, CHF, hypertension resistant to treatment, Type 2 DM, stroke, pulmonary hypertension, high-risk driving population) to be high risk for SDB and recommends sleep apnea symptom evaluation (not equivalent to polysomnography) (33). However, even screening in these high-risk groups is not carried out widely.

In practice, excessive daytime sleepiness or snoring is what leads to screening and perhaps eventually polysomnography. Providers may refer for diagnostic screening if patients snore and have other clinical features of OSA including symptomatology (witnessed apnea, frequent awakening, cognitive deficits, morning headaches, etc.), and other clinical findings, such as obesity, hypertension, cardiac dysrhythmia, cardiovascular, or cerebrovascular disease. The ORBIT – AF trial found an 18% frequency of OSA (defined by clinician defined baseline diagnosis and CPAP treatment) in a cohort of patients with AF. Likely, patients with AF will have other risk factors and symptoms that lead to OSA screening. A methodical analysis of screening for OSA in all AF patients would be of interest, given that an effective relatively low-cost intervention exists to treat OSA. Additionally, it is known that OSA can affect the efficacy of treatments and recurrence of AF. Thus, it seems reasonable to conduct OSA screening in patients with AF with risk factors to ensure optimization of AF treatment strategies. This includes screening in patients with AF and history of stroke. Screening for OSA in patients who develop recurrent AF after undergoing an ablation procedure is also reasonable. Treatment of OSA along with risk factor modification would presumably improve treatment of AF and result in better control of risk factors that predispose to stroke.

## Conclusion

The common mechanisms linking OSA and AF are complex and mediated by many mechanisms. Animal and human studies have demonstrated that the pathophysiologic changes brought on by sleep apnea, including changes in intrathoracic pressure, hypoxia, and hypercapnia may cause structural and electrical changes that predispose to arrhythmia including AF.

Further research into the benefits of screening patients with OSA for AF, and *vice versa* is needed. The reduction in complications, and thus health care savings from optimizing care for those diagnosed with OSA and AF, could potentially outweigh costs of screening.

Obstructive sleep apnea has been shown to be an independent risk factor for stroke and may increase the rate of AF. While AF is a known cause of stroke, currently research into other cardiac conduction abnormalities that may contribute to stroke is ongoing and in the future may better inform this relationship. Current knowledge demonstrates that the interplay between OSA and AF as contributors to stroke is multidirectional. The direct and indirect cost associated with stroke care imposes a huge financial burden both on an individual and societal level. Effective and relatively low-cost therapies exist to address OSA and AF. Early detection of OSA and AF may lead to initiation of appropriate treatment such as anticoagulation and may represent a novel method to reduce the stroke burden, and ultimately may save health care dollars.

The ideal methods for early OSA detection in at risk patients require further study. Untreated severe OSA is associated with increased cardiovascular mortality of any cause, and thus OSA should be aggressively managed with CPAP. Additionally, use of CPAP can reduce AF recurrence. Thus, in patients with OSA and AF, CPAP use should be optimized to reduce AF recurrence and improve results of AF treatment.

Although screening, diagnosis, and treatment are crucial for both AF and OSA in terms of alleviating associated morbidity and mortality, as with any cardiovascular disease, prevention is the key. For some patients, physical activity and weight loss can significantly decrease AF burden (34, 35) and OSA severity. Taking control of this modifiable risk factor will likely have a pronounced effect on the prevalence of these two linked disorders. For patients who already suffer from AF, OSA, or both, the importance of lifestyle modifications to combat obesity and improve overall cardiovascular health cannot be overstated and should be a major focus of treatment.

## Author Contributions

EM-L wrote the first draft. SC provided references, critical revisions to the manuscript, and details regarding ongoing trials related to the topic.

## Conflict of Interest Statement

EM-L has nothing to disclose. SC received funding from Boehringer Ingelheim and is principal investigator for the ADIOS study. The handling editor declared a shared affiliation, though no other collaboration, with the authors SC and EM-L.
